# Revealing the Saline Adaptation Strategies of the Halophilic Bacterium *Halomonas beimenensis* through High-throughput Omics and Transposon Mutagenesis Approaches

**DOI:** 10.1038/s41598-017-13450-9

**Published:** 2017-10-12

**Authors:** Yan-Huey Chen, Chia-Wei Lu, Yuan-Tay Shyu, Shih-Shun Lin

**Affiliations:** 1 Department of Horticulture and Landscape Architecture, National Taiwan University, Taipei, 106 Taiwan; 2 Institute of Biotechnology, National Taiwan University, Taipei, 106 Taiwan; 3 Agricultural Biotechnology Research Center, Academia Sinica, Taipei, 115 Taiwan; 4 Center of Biotechnology, National Taiwan University, Taipei, 106 Taiwan; 5 National Center for High-Performance Computing, National Applied Research Laboratories, Hsinchu, 300 Taiwan

## Abstract

Studies on the halotolerance of bacteria are attractive to the fermentation industry. However, a lack of sufficient genomic information has precluded an investigation of the halotolerance of *Halomonas beimenensis*. Here, we describe the molecular mechanisms of saline adaptation in *H. beimenensis* based on high-throughput omics and Tn5 transposon mutagenesis. The *H. beimenensis* genome is 4.05 Mbp and contains 3,807 genes, which were sequenced using short and long reads obtained via deep sequencing. Sixteen Tn5 mutants with a loss of halotolerance were identified. Orthologs of the mutated genes, such as *nqrA*, *trkA*, *atpC*, *nadA*, and *gdhB*, have significant biological functions in sodium efflux, potassium uptake, hydrogen ion transport for energy conversion, and compatible solute synthesis, which are known to control halotolerance. Other genes, such as *spoT*, *prkA*, *mtnN*, *rsbV*, *lon*, *smpB*, *rfbC*, *rfbP*, *tatB*, *acrR1*, and *lacA*, function in cellular signaling, quorum sensing, transcription/translation, and cell motility also shown critical functions for promoting a halotolerance. In addition, KCl application increased halotolerance and potassium-dependent cell motility in a high-salinity environment. Our results demonstrated that a combination of omics and mutagenesis could be used to facilitate the mechanistic exploitation of saline adaptation in *H. beimenensis*, which can be applied for biotechnological purposes.

## Introduction

Halotolerant and halophilic bacteria have the ability to adapt to high salt concentrations. This trait can be exploited by the fermentation industry to conserve energy and water^1^. Due to their specific mechanisms of saline adaptation, halotolerant bacteria can tolerate high-salinity stress, whereas halophilic bacteria require a certain saline concentration to survive^2^. Several molecular approaches to saline adaptation have been discovered in bacteria, including the accumulation of compatible solutes, potassium uptake, and sodium effluxion^3,4^. Compatible solutes (*e.g*. betaine, ectoine, glutamate, trehalose, and proline) are low molecular weight compounds that are synthesized by bacteria to maintain cellular turgidity and electrolyte concentrations^4^. The role of compatible solutes overlaps with that of potassium uptake in the maintenance of osmotic equilibrium. When *Escherichia coli* (a non-halotolerant bacterium) is exposed to saline stress, potassium concentration first transiently increases and then rapidly decreases after the *de novo* synthesis of compatible solutes, indicating that the osmoprotective role of these compatible solutes supplants that of potassium^3^. By contrast, the potassium and glutamate concentrations of *Halomonas elongata* (a halotolerant bacterium) continue to increase even after ectoine is generated, suggesting that the halotolerant of *E. coli* and *H. elongata* differ^3^.

In *Halomonas* spp., potassium uptake and organic compatible solute synthesis controls respiratory chain and osmotic regulation under high-salinity conditions^5^. Trk, Ktr, and Kdp are three major systems of potassium uptake in bacteria^3^. The Trk system requires ATP and drives potassium uptake through the transmembrane electrochemical proton gradient^3^. TrkA of *E. coli*, which is involved in the Trk system, contains a nicotinamide adenine dinucleotide (NAD^+^)-binding domain that can bind NAD^+^ or NADH^6^. Moreover, a *trkA* mutant of *H. elongata* showed defects in potassium transportation^3^. The Ktr system is a Na^+^-dependent potassium uptake system, whereas the Kdp system is classified as a P-type K^+^-translocating ATPase that hydrolyzes ATP to generate the driving force for K^+^ uptake^3,7^. Therefore, sodium ion efflux and hydrogen and potassium ion uptake help maintain bacterial osmotic balance in high-salinity environments.

In general, bacteria maintain high intracellular potassium and low sodium concentrations for effective cellular enzyme activity, and some marine bacteria may maintain a sodium gradient for energy metabolism^8,9^. To prevent entry of sodium into the cell, the sodium-translocating NADH:quinone oxidoreductase (Na^+^-NQR) uses exergonic energy to pump hydrogen or sodium ions from the cytosol to the periplasm through symporters^9^. In *Vibrio cholera*, NqrA is a subunit of Na^+^-NQR that acts as a sodium pump and can bind quinone^9,10^. The operation of the respiratory chain generates an electrochemical sodium gradient to pump out sodium to maintain osmotic balance while NADH is oxidized by Na^+^-NQR^9^. Under high-salinity conditions, bacteria maintain a low internal sodium concentration via Na^+^/H^+^ antiporters^9^.

Cell motility and energy production also play critical roles in halotolerance^11,12^. Flagellum-related genes have been found to be down-regulated in highly saline environments, suggesting that decreased cell motility allows more energy to be available for osmoprotection^11,12^. Another explanation is that cell motility is correlated with sodium-driven motor activity^11^. Therefore, decreased motor activity can reduce sodium re-entry to maintain intracellular ion homeostasis^11^.

Currently, complete genomic sequence are available for approximately 6,892 of the 87,815 bacterial genomes in NCBI (2016), including 7 *Halomonas* spp. Microarray and next-generation sequencing (NGS) approaches have been used to examine transcriptome profiles related to saline adaptation in *Bacillus* sp., *Desulfovibrio vulgaris*, *Staphylococcus* sp., *Mesorhizobium alhagi*, and *Jeotgalibacillus malaysiensis*, and the alkaline response of *Halomonas* sp.^1,12–15^ Moreover, mutant lines have been generated by transposon mutagenesis to study halotolerance in several bacteria, including *Caulobacter crescentus*, *Sinorhizobium fredii*, *Azospirillum brasilense*, and *Sinorhizobium meliloti* ^16–19^. Moreover, Kindzierski *et al*. (2017) integrated metabolome and proteome analyses to study the osmoregulation of *H. elongata* and demonstrated that glycolysis- and tricarboxylic acid cycle-related enzymes are involved in compatible solute biosynthesis for osmoregulation^20^.

In this study, we isolated the NTU-111 strain of *Halomonas beimenensis* (hereafter referred to as *H. beimenensis*) and sequenced its entire genome using NGS. *H. beimenensis* gene expression profiles under various salt conditions were also analyzed by transcriptome analysis. Moreover, we identified 16 Tn5 insertion mutants that had lost the ability to tolerate 15% NaCl. Based on these studies, we propose a conceptual model for *H*. *beimenensis* adaptation to a high-salinity environment.

## Results

### *H. beimenensis* growth conditions and phylogenetic position

**Figure 1:**
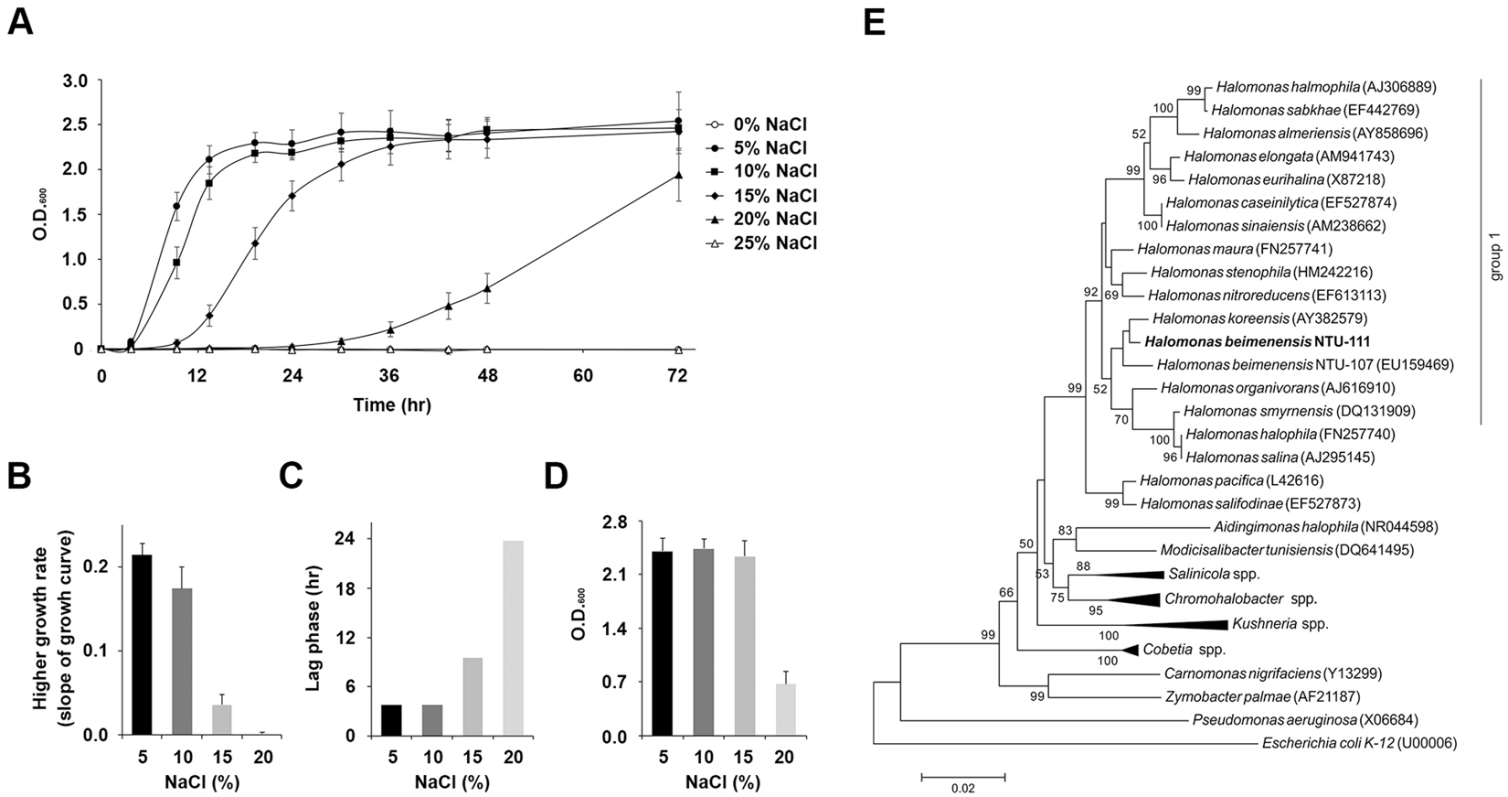
Phylogenetic position and growth conditions of *Halomonas beimenensis*. (**A**) Growth curve of *H. beimenensis* in medium containing various concentrations of NaCl, including 0%, 5%, 10%, 15%, 20%, and 25% (w/v) NaCl. (**B**) The slope of growth curve in the 5%, 10%, 15%, and 20% NaCl conditions between 3 and 12 h represents the growth rate of *H. beimenensis* under different NaCl conditions. (**C**) The length of the lag phase of *H. beimenensis* under different NaCl conditions. (**D**) The concentration of *H. beimenensis* (OD_600_) under different NaCl conditions at 48 h. (**E**) A phylogenetic tree based on 16S rRNA sequences was constructed by the neighbor-joining method with the Juke-Cantor correction. Bootstrap values were calculated from 1,000 samplings, and values > 50% are shown. Bar, 0.02 substitutions per nucleotide position.

*H. beimenensis* requires at least 5% NaCl to survive, and no bacterial growth was observed in the presence of 0% NaCl condition (Fig. 1A). The growth curves revealed a higher growth rate in 5% NaCl (0.21) than in 10% (0.19), 15% (0.04), and 20% (0.0003) NaCl between 3 and 12 h, whereas no detectable growth was observed in 25% NaCl during this time (Fig. 1B). We also calculated the length of the lag phase of *H. beimenensis* under various NaCl conditions. The *H. beimenensis* lag phase in 5% and 10% NaCl was 3.8 h, whereas this phase in 15% and 20% NaCl lasted 9.5 and 23.7 h, respectively (Fig. 1C). In addition, *H. beimenensis* growth was inhibited at >20% NaCl (Fig. 1A). *H. beimenensis* has a fast growth rate in 5% NaCl; however, the maximal biomass yield was not significantly different in 5%, 10%, and 15% NaCl at 48 h (Fig. 1D). These results indicated that the optimal growth condition for *H. beimenensis* is 5% NaCl. Moreover, *H. beimenensis* exhibits halotolerance at concentrations of 10 to 20% NaCl and can thus be classified as a moderately halophilic bacterium.

In a neighbor-joining-based phylogenetic tree of 16S ribosomal RNAs (rRNAs), *H. beimenensis* was clustered into group 1^21^ (Fig. 1E). The mean identity for group 1 was 96.92%. In the phylogenetic tree, *H. beimenensis* was closest to *H. koreensis* (99.1% identity), the NTU-107 strain of *H. beimenensis* (98.8% identity), and *H. organivorans* (98.7% identity). NTU-111 and NTU-107 were isolated from the same location, and the phylogenetic tree results agree with the results published by Wang *et al*.^22^. In addition, identities of 92.3–96.4% were found when comparing *H. beimenensis* to the other *Halomonas* spp. in group 1 (Fig. S1, and Supplementary Table S1). In contrast, the mean 16S rRNA identity between *H. beimenensis* and other genera was less than 95% (Fig. 1E, Supplementary Fig. S1, and Supplementary Table S1). The phylogenetic data indicated that *H. beimenensis* is a new strain of *Halomonas* spp.

### Genomic features and transcriptome profiles of *H. beimenensis*

**Figure 2:**
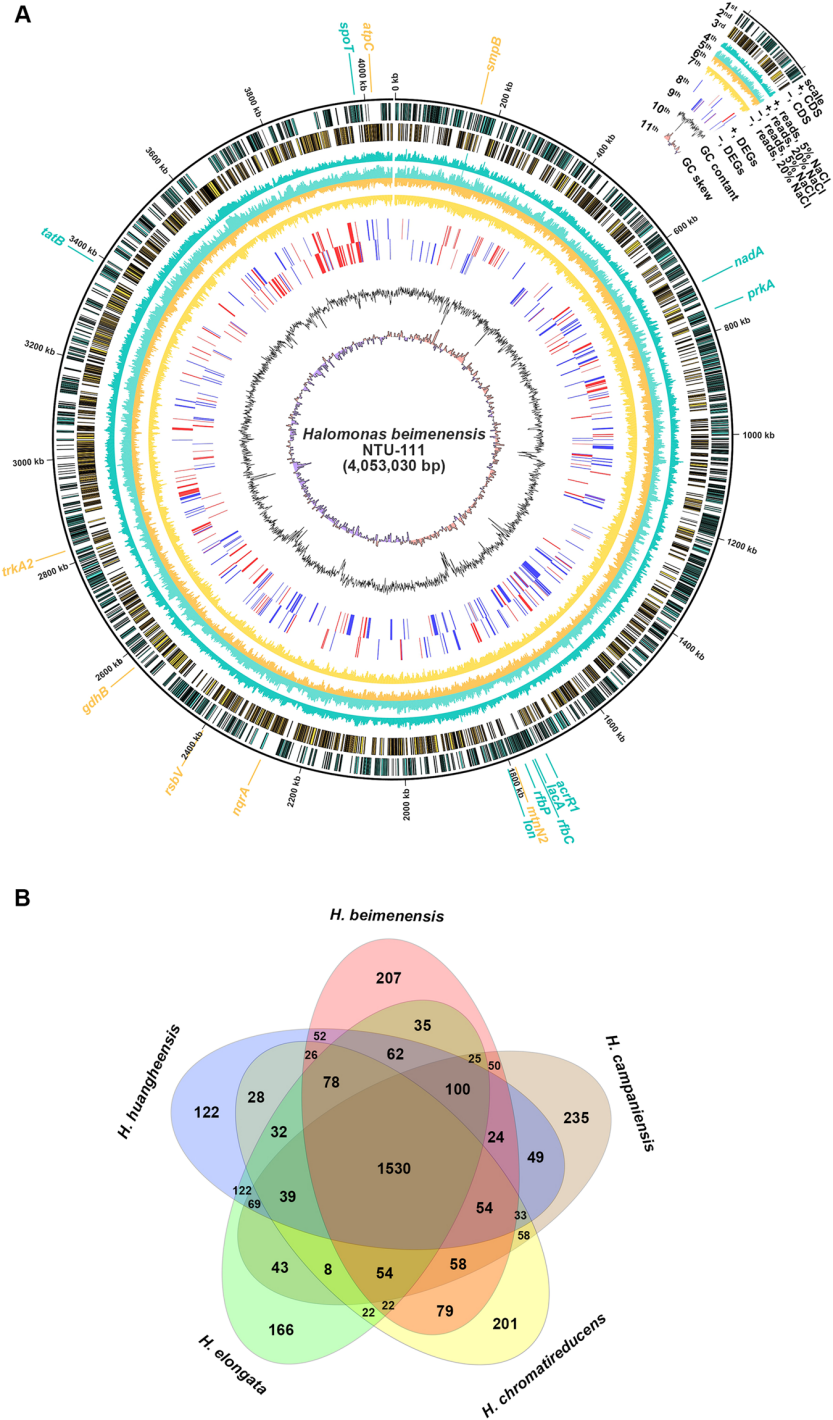
Genome map and gene comparison of *Halomonas beimenensis*. (**A**) Genome map of *H. beimenensis*. Rings from the outside are as follows: 1^st^ circle: scale marks (unit, kb); 2^nd^ circle: predicted genes on the + strand; 3^rd^ circle: predicted genes on the − strand; 4^th^ circle: read counts mapping to predicted genes on the + strand in 5% NaCl; 5^th^ circle: read counts mapping to predicted genes on the + strand in 20% NaCl; 6^th^ circle: read counts mapping to predicted genes on the - strand in 5% NaCl; 7^th^ circle: read counts mapping to predicted genes on the − strand in 20% NaCl; 8^th^ circle: significantly differentially expressed genes determined based on log_2_ fold-changes (log_2_FC) in FPKM values on the + strand that were greater than 2 (up-regulated genes, red lines) or smaller than −2 (down-regulated genes, blue lines); 9^th^ circle: significantly differentially expressed genes determined based on log_2_FC in FPKM values on the - strand greater than 2 (up-regulated gene, red lines) or smaller than -2 (down-regulated genes, blue lines); 10^th^ circle: GC content, 11^th^ circle: GC skew (above-average values in pink; below-average values in purple). (**B**) Venn diagram of gain and loss of genes between *H*. *beimenensis* and four *Halomonas* spp.: *H*. *campaniensis*, *H*. *chromatireducens*, *H*. *elongata*, and *H*. *huangheensis*.

The entire sequence of the *H. beimenensis* genome was hybrid *de novo* assembled with reads from Illumina MiSeq (3,719,926 reads) and PacBio SMRT (39,996 reads) by SPAdes Genome Assembler V3.5.0^23^. One large contig of 4,053,030 bp was obtained, which contained 199 bp regions at the 5′- and 3′-ends that overlapped exactly, indicating that the contig could be completely circularized (GenBank CP021435) (Fig. 2A). A total of 3,807 identified coding sequences (CDSs) were spread across the positive strand (1,868 CDSs) and negative strand (1,939 CDSs) (Fig. 2A). The average G + C content was 68.4%, and the GC skew indicated that the replication origin and replication terminus were located at 65 kb and 2,075 kb (Fig. 2A). Gene Ontology (GO) and Clusters of Orthologous Groups (COG) categories are provided in Supplementary Figures S2 and S3.

A total of 1,530 ortholog genes were detected in *Halomonas campaniensis*, *H*. *chromatireducens*, *H*. *elongata*, and *H*. *huangheensis* (Fig. 2B). *H. beimenensis* contains 207 unique genes and lacks 1,326 genes that are present in the other 4 *Halomonas* spp. (Fig. 2B). The transcriptome profiles of *H. beimenensis* grown in 5% and 20% NaCl were used to identify differentially expressed genes (DEGs). The transcriptome reads obtained from bacteria grown in 5% (5,070,041 reads) and 20% (6,154,708 reads) NaCl were compared to the genomic sequence to determine the mapping rate and to perform fragments per kilobase of transcript per million mapped reads (FPKM) calculations. The mapped transcriptome reads are shown in the genome map (Fig. 2A). The 4^th^ and 6^th^ circles represent the transcriptome read counts on the + and − strands in the 5% NaCl condition, respectively, whereas the 5^th^ and 7^th^ circles represent the transcriptome read counts on the + and − strands in the 20% NaCl condition, respectively (Fig. 2A). Our data indicated that approximately 98.66% (5% NaCl) and 96.02% (20% NaCl) of the transcriptome reads were mapped to the genomic sequence.

**Figure 3:**
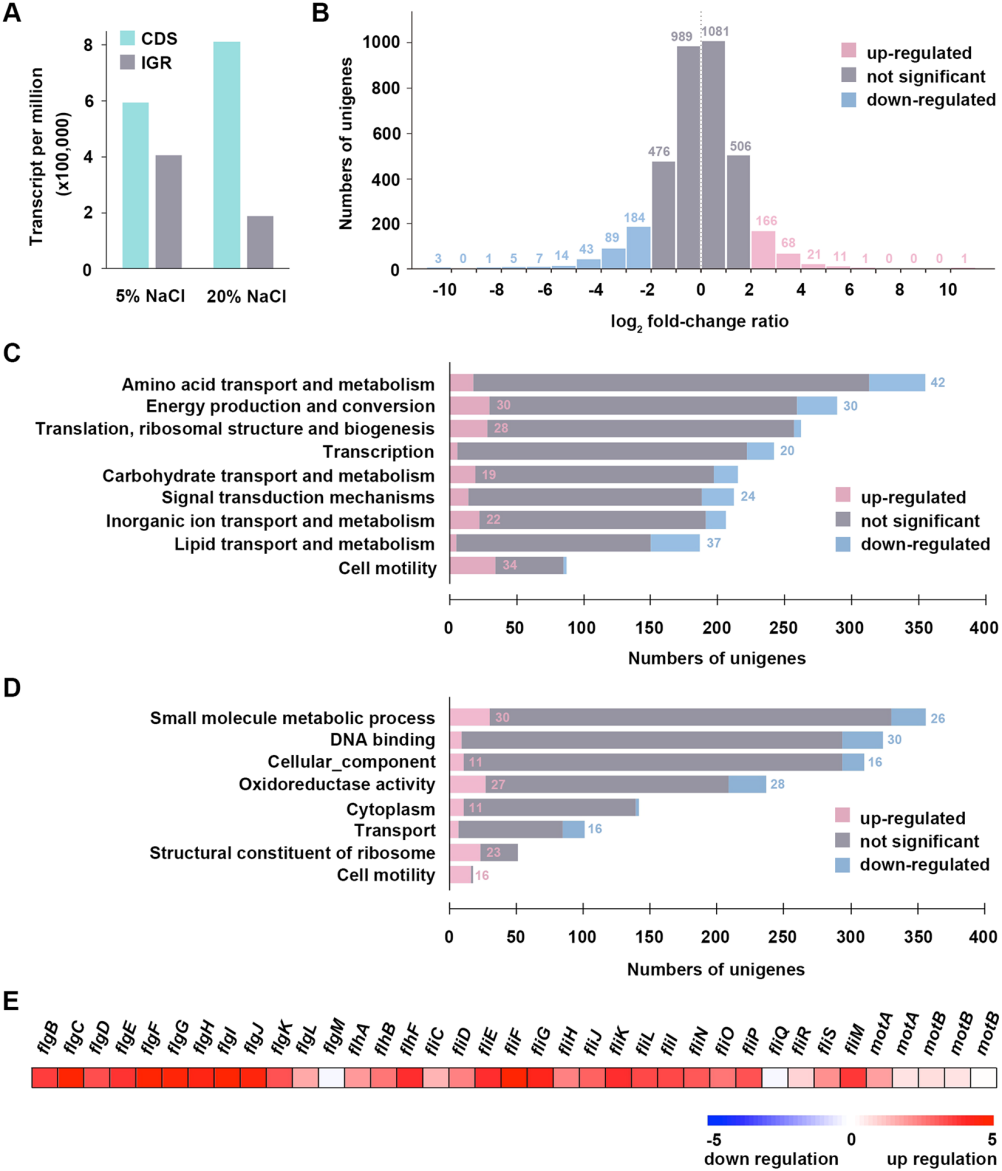
Gene features of *Halomonas beimenensis*. (**A**) The average TPM (transcripts per million reads) of CDSs and the intergenic regions in the 5% and 20% NaCl conditions. (**B**) The differentially expressed genes (DEGs) of *H. beimenensis* between the 5% NaCl and 20% NaCl conditions. The pink columns represent the up-regulated DEGs, for which the log_2_FC of FPKM was higher than 2; the blue columns represent the down-regulated DEGs, for which log_2_FC in FPKM was lower than −2; the gray columns represent genes that did not significantly change (the absolute value of the log_2_FC of FPKM did not exceed 2). Numbers above the column represent the numbers of genes. (**C**) The top 5 clusters of orthologous groups (COG) categories indicated for the DEGs of *H. beimenensis*. (**D**) The top 8 clusters of Gene Ontology (GO) categories indicated for the DEGs of *H. beimenensis*. Up-regulated DEGs (log_2_FC of FPKM > 2) are represented by pink bars and numbers; the down-regulated DEGs (log_2_FC of FPKM < −2) are represented by blue bars and numbers; gray bars indicate genes with no significant change (−2 < log_2_FC of FPKM < 2). (**E**) The transcriptome profile of differentially expressed flagella-related genes in 5% and 20% NaCl.

Approximately 53.61% (in 5% NaCl) and 74.59% (in 20% NaCl) of the reads mapped to CDSs, whereas 40.05% (in 5% NaCl) and 21.43% (in 20% NaCl) of the reads mapped to intergenic regions (IGRs), implying that intergenic regions of the genome can also generate RNA transcripts. In 5% NaCl, the average TPM (transcripts per million reads) of CDSs and IGRs was approximately 594,952.57 and 405,047.43, respectively; in 20% NaCl, the average TPM of CDSs and IGR was approximately 811,185.16 and 188,814.83, respectively (Fig. 3A). These data suggest that CDSs exhibit higher transcriptional expression than intergenic regions and that under the high saline condition, more reads mapped to CDSs regions than to IGR regions.

We identified 614 DEGs (268 up-regulated genes and 346 down-regulated genes) showing a log_2_ fold-change (log_2_FC) in FPKM >2 between the 5% and 20% NaCl conditions (Fig. 3B and Table S2). The top COG and GO categories, with the corresponding number of DEGs, are highlighted in Fig. 3C and D, respectively. The COG data indicated that 34 DEGs related to “cell motility”, 30 DEGs related to “energy production and conversion”, and 22 DEGs related to “inorganic ion transport and metabolism” were up-regulated in 20% NaCl, whereas 30 DEGs related to “energy production and conversion” were down-regulated under such conditions (Fig. 3C and Supplementary Table S2). Moreover, the GO category analysis revealed that 16 DEGs related to “cell motility” were up-regulated (Fig. 3D). Therefore, cell motility plays an important role in halotolerance. In fact, many flagellum-related *H. beimenensis* genes were up-regulated in 20% NaCl (Fig. 3E). Detailed information on the genes corresponding to various COG and GO categories is provided in the Supplementary material. The transcriptome profiles of *H. beimenensis* in 5% and 20% NaCl are available at the ContigViews database (www.contigviews.bioagri.ntu.edu.tw).

### Halotolerance-related genes

**Figure 4:**
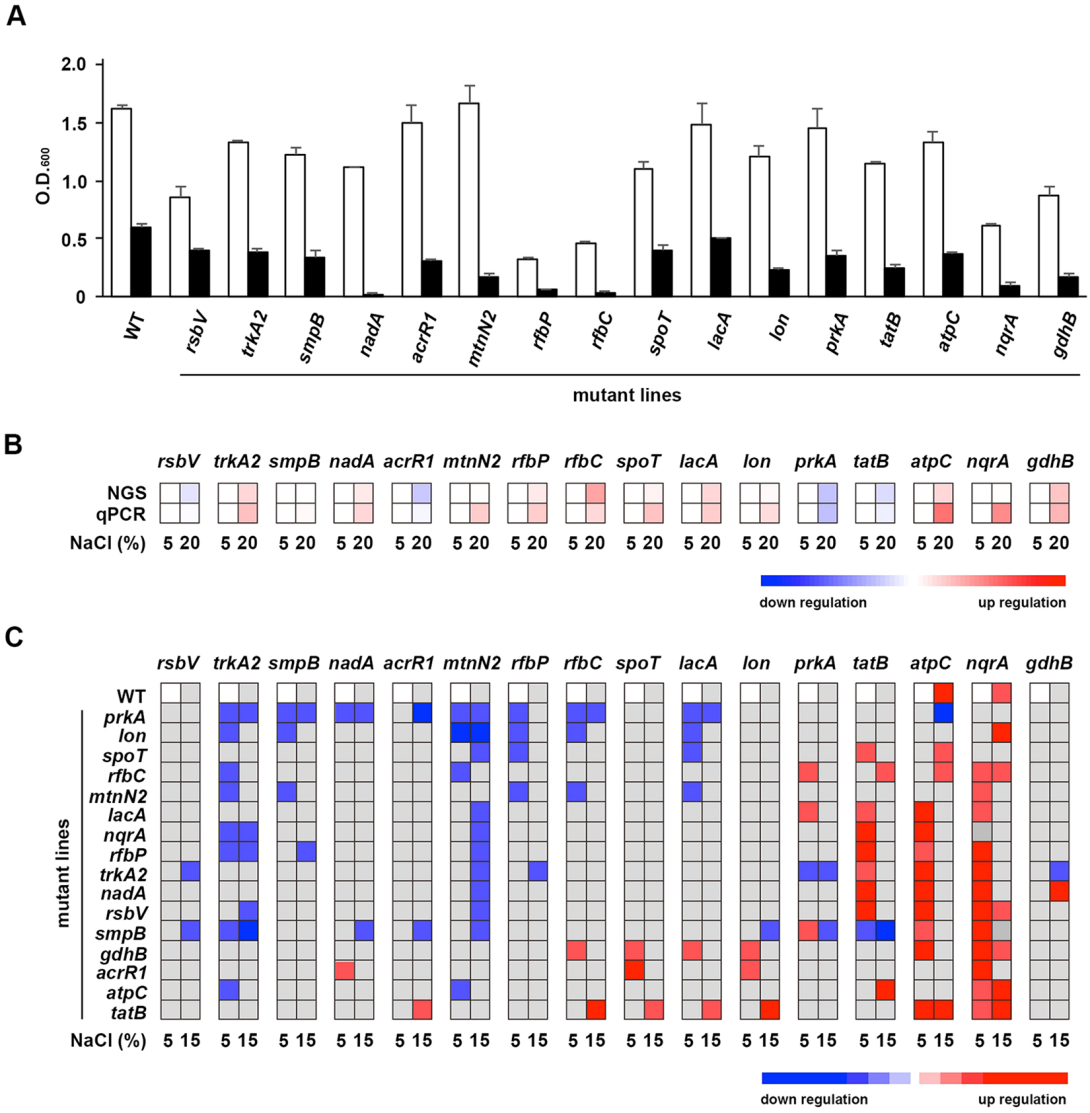
Growth and gene expression levels of 16 halotolerance-related genes in the wild-type and Tn5 mutants of *Halomonas beimenensis* under different salt conditions. (**A**) The growth of wild-type (WT) *H. beimenensis* and 16 Tn5 mutant lines under 5% and 15% NaCl conditions. (**B**) The expression of 16 halotolerance-related genes in WT *H. beimenensis* was determined by the FPKM values from the transcriptome data and qRT-PCR analysis of bacteria grown in 5% and 20% NaCl conditions. (**C**) The highlighted gene expression of the 16 genes in the WT bacteria and the 16 Tn5 mutants of *H. beimenensis* under 5% and 15% NaCl conditions analyzed by qRT-PCR. Each data point was compared with the gene expression in WT bacteria grown in 5% NaCl.

Of the 1,256 Tn5 transposition clones, 22 showed a loss of or decreased tolerance to 15% NaCl. In these 22 clones, 16 unique genes were interrupted by the Tn5 transposon (Fig. 4A and Fig. S4). The transcriptome profile showed that 11 out of these 16 genes were up-regulated in 20% NaCl, including the potassium transporter gene *trkA2*, the sodium-translocating NADH:quinone oxidoreductase gene *nqrA*, the ATP synthase gene *atpC*, the NAD-specific glutamate dehydrogenase gene *gdhB*, the quinolinate synthetase gene *nadA*, the dTDP-4-dehydrorhamnose 3,5-epimerase gene *rfbC*, the undecaprenyl-phosphate galactosephosphotransferase gene *rfbP*, the (p)ppGpp synthetase/guanosine-3′,5′-bis(diphosphate) 3′-diphosphatase gene *spoT*, the ATP-dependent protease gene *lon*, the 5′-methylthioadenosine/S-adenosylhomocysteine nucleosidase gene *mtnN2*, and the acetyltransferase gene *lacA*, whereas the anti-anti-sigma factor gene *rsbV*, the *PrkA* family serine protein kinase gene *prkA*, the twin-arginine translocation protein gene *tatB*, and the *TetR* family transcriptional regulator gene *acrR1* were down-regulated. Notably, *lon*, *mtnN2*, and *nqrA* showed only a slight up-regulation in the transcriptome profiles (Fig. 4B, upper panel).

We used quantitative reverse transcription polymerase chain reaction (qRT-PCR) to verify the expression of the 16 genes, and the obtained data agreed with the NGS transcriptome profile (Fig. 4B and C). Notably, the tmRNA-binding protein gene *smpB* did not exhibit significantly different expression between the 5% and 20% NaCl conditions (Fig. 4B). In the COG classifications, among the 16 genes, the *atpC*, and *nqrA* were in the “energy production and conversion” category, whereas *trkA2* was in the “inorganic ion transport and metabolism” category. *rfbP* and *rfbC* were in the “cell wall/membrane/envelope biogenesis” category, whereas *gdhB* was in the “amino acid transport and mechanisms”. By contrast, the *nadA*, and *spoT* were categorized as the “small molecule metabolic process” category according to GO, whereas *prkA*, *rsbV*, and *spoT* were categorized under the “signal transduction mechanisms” category according to COG. *acrR1* was in the “transcription” category according to COG and the “DNA binding” category according to GO. *lon* and *smpB* were in the “posttranslational modification, protein turnover, chaperones” category according to COG, implying these genes play roles in protein translation. The COG and GO categories of the remaining three genes did not indicate clear biological functions. Interestingly, previous studies have demonstrated that AtpC, NqrA, and TrkA2 are involved in energy production and potassium uptake, whereas NadA is involved in compatible solute biosynthesis^3,10,24–26^. Mutation of the *trkA* and *nqr* genes in *H. elongata* and *V. cholerae*, respectively, results in loss of halotolerance^3,27^. Therefore, these results were confirmed by examining the halotolerance of the Tn5 mutants, and it was indicated that energy production and ion transportation play important roles in halotolerance. The COG and GO categories for the 16 genes are listed in Supplementary Table S3.

### Gene expression profiles of the Tn5 mutant lines

To study the adaptation of the 16 Tn5 mutants that exhibited gene expression differences in 5% and 20% NaCl, we evaluated gene expression in wild-type (WT) *H. beimenensis* and the 16 Tn5 mutants grown in 15% NaCl by qRT-PCR (Fig. 4C). To highlight the importance of these 16 genes in halotolerance regulation, each gene was assigned a specific cut-off threshold based on the significant *p*-value (Table S4) for up- or down-regulation compared with the gene expression in the WT at 5% NaCl, and these expression patterns are shown in Fig. 4C.

With the exception of *gdhB* gene, the remaining 15 genes could be classified as “positive”, “negative”, or “bifunctional” regulatory genes in the halotolerant response. In some cases, the gene mutation did not affect the expression of other genes, but any gene that was affected by mutation of another gene was classified as a “regulated gene”. For instance, mutation of *prkA* resulted in the down-regulation of many other genes in 5% and 15% NaCl, suggesting that *prkA* is a positive regulatory gene (Fig. 4C). By contrast, *gdhB* and *tatB* mutations led to many genes being up-regulated in 5% and 15% NaCl, suggesting that these two genes are negative regulatory genes (Fig. 4C). The *spoT* was classified as a bifunctional regulatory gene because the mutant caused the up- or down-regulation of various genes in 5% and 15% NaCl (Fig. 4C).

In 5% NaCl, *lon* was classified as a positive regulatory gene, whereas *nadA*, *rsbV*, *acrR1*, and *lacA* were classified as negative regulatory genes (Fig. 4C). Moreover, *rfbP*, *nqrA*, *mtnN2*, *atpC*, *rfbC*, *smpB*, and *trkA2* were classified as bifunctional regulatory genes (Fig. 4C). However, the genes originally observed to be positive or negative regulatory genes in 5% NaCl might play different roles in 15% NaCl to trigger halotolerance. For instance, *rfbP*, *nqrA*, *lacA*, *smpB*, and *trkA2* were classified as positive regulatory genes, whereas *rfbC* and *atpC* were classified as negative regulatory genes in 15% NaCl (Fig. 4C). Moreover, *nadA*, *rsbV*, and *lon* were classified as bifunctional genes, whereas *mtnN2* and *acrR1* were classified as regulated genes (Fig. 4C).

**Figure 5:**
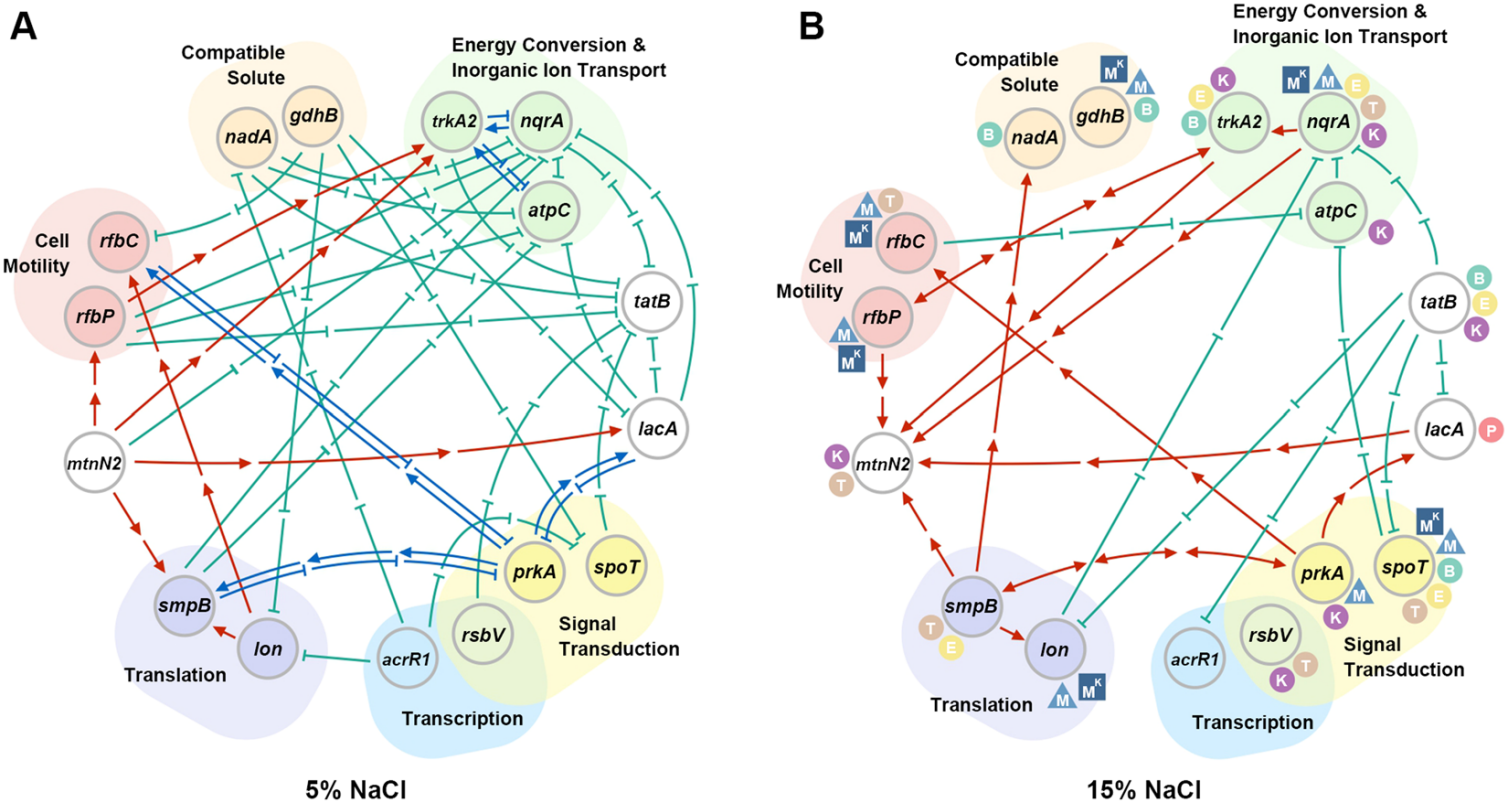
The network of 16 halotolerance-related genes of *Halomonas beimenensis*. (**A**) The putative network in the 5% NaCl condition. (**B**) The putative network in the 15% NaCl condition. The symbols for each gene suggest involved functions, *e.g*., (K) potassium; (B) betaine; (E) ectoine; (P) proline; (T) trehalose; (M) cell motility; and (M^K^) potassium-dependent cell motility.

According to the gene expression observed in the WT and mutants, we constructed gene-for-gene networks to summarize the relationships between these critical genes (Fig. 5). The regulatory network of these 16 genes was more complex in 5% NaCl (Fig. 5A) than in 15% NaCl (Fig. 5B). Regarding the promotion of gene expression, MtnN2 promoted *rfbP*, *trkA2*, *smpB*, and *lacA* expression, whereas Lon promoted *smpB* and *rfbC* expression in 5% NaCl (Fig. 5A). Moreover, RfbP promoted *trkA2* expression (Fig. 5A). In contrast, most of the examined genes exhibited complex negative regulatory relationships in 5% NaCl (Fig. 5A). *nqrA*, *atpC*, and *tatB* were the most negatively regulated genes in the 5% NaCl condition (Fig. 5A). Interestingly, *prkA* exhibited feedback control with *smpB*, *lacA*, and *rfbC* (Fig. 5A). *trkA2* also exhibited feedback with *atpC* and *nqrA* (Fig. 5A).

Surprisingly, most of the negative relationships observed in the 5% NaCl condition were not active in 15% NaCl (Fig. 5B). There are several important changes that must be highlighted. First, in 15% NaCl, MtnN2 ceased promoting expression of the other examined genes, and its expression was promoted by RfbP, NqrA, LacA, SmpB, and TrkA2 (Fig. 5B). Second, TatB acted as an indirect repressor to negatively affect *nqrA*, *lon*, *lacA*, *spoT*, and *acrR1* expression, whereas *tatB* was negatively regulated in 5% NaCl (Fig. 5B). TatB is involved in the translocation of folded proteins across the cytoplasmic membrane^28^; therefore, loss of its function might have consequences for many aspects of cell metabolisms. Third, several genes involved in transcription/translation and signaling affected the expression of genes related to energy conversion and inorganic ion transport, compatible solute or translation in 15% NaCl. For instance, SpoT and RfbC specifically affected *atpC*, and SmpB promoted the expression of *nadA* and *lon* (Fig. 5B). Finally, *trkA2* exerted a bilateral promotion effect together with *rfbP* (Fig. 5B). These dynamic variations suggest adaptation to high-salinity conditions.

### Chemical growth complementation of the Tn5 mutants

**Figure 6:**
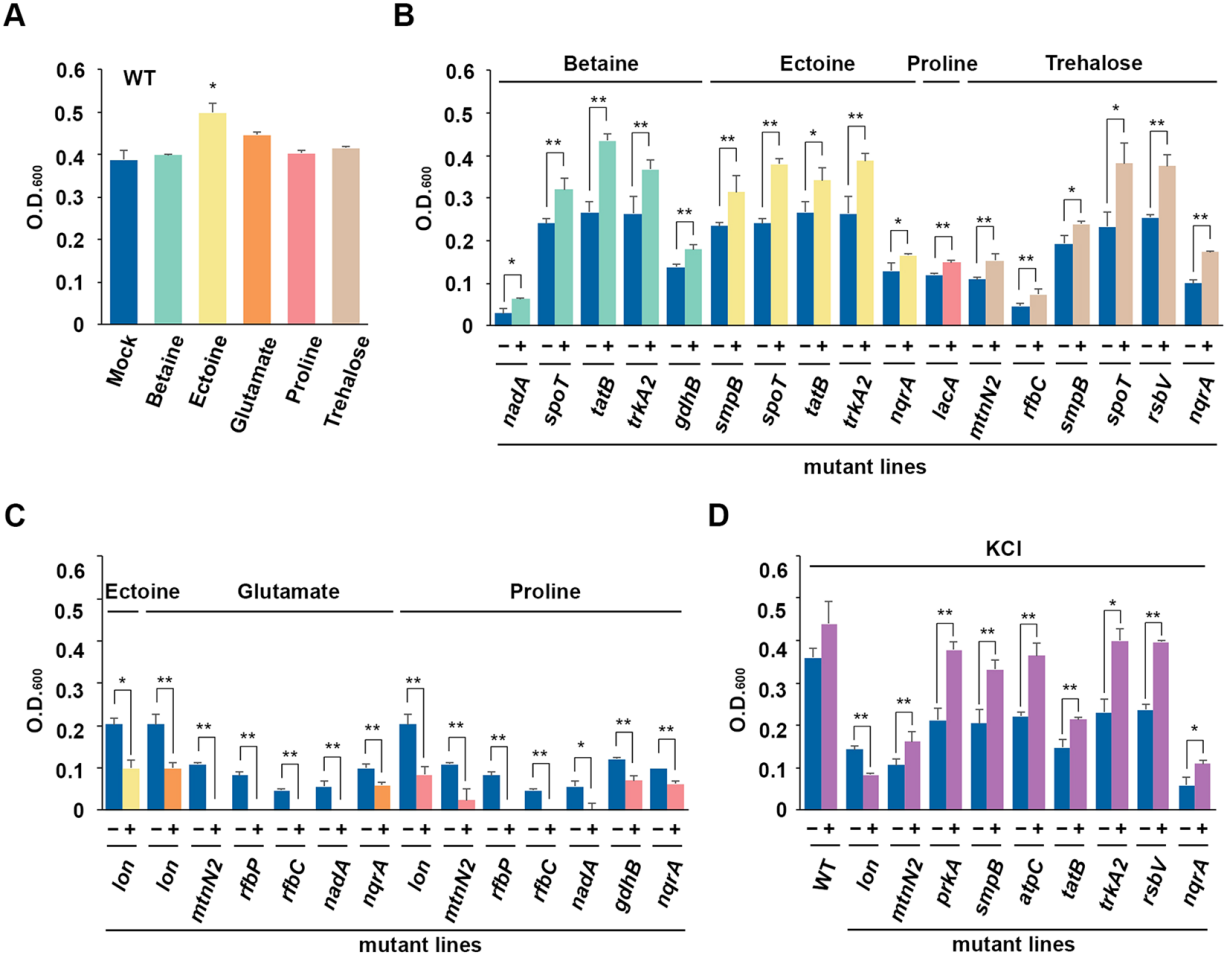
Chemical complementation of wild-type and Tn5-mutated *Halomonas beimenensis* in 15% NaCl. (**A**) The complementation analysis of the wild-type bacteria with a variety of compatible solutes. (**B**) The growth of Tn5 mutant lines in which the mutant phenotype was slightly or fully rescued by a variety of compatible solutes. (**C**) The growth of Tn5 mutant lines that were inhibited by a variety of compatible solutes. (**D**) The growth of Tn5 mutant lines in which the mutant phenotype was slightly or fully rescued by 200 mM KCl.

Accumulations of KCl and organic compatible solutes is a critical approach used by halophilic bacteria to overcome lower external water activity under high-salinity conditions^29^. We hypothesized that some of the Tn5 mutants may have lost the ability to maintain ionic strength or to regulate the biosynthesis of organic compatible solutes. Therefore, we examined whether the application of additional KCl and compatible solutes could help Tn5 mutants to adapt to high-salinity stress. In WT *H. beimenensis*, treatment with 20 mM ectoine, 20 mM glutamate, and 200 mM KCl slightly enhanced bacterial growth, whereas the other compatible solutes did not affect *H. beimenensis* growth (Fig. 6).

Treatment with 20 mM betaine and 20 mM ectoine compensated for the loss of halotolerance in the *spoT*, *tatB*, and *trkA2* mutants grown in 15% NaCl, resulting in growth similar to that of WT bacteria (Fig. 6A and B, and Table 1). Moreover, 20 mM ectoine can compensate for an *smpB* mutation (Fig. 6A and B, and Table 1). For the *nadA* and *gdhB* mutants, betaine only slightly rescued the mutant phenotype in 15% NaCl (Fig. 6B and Table 1). Treatment with 20 mM trehalose fully rescued the *spoT* and *rsbV* mutants but only slightly compensated for the *rfbC*, *smpB*, *mtnN2* and *nqrA* mutations (Fig. 6A and B, and Table 1). However, 20 mM glutamate and 20 mM proline inhibited rather than rescued growth in the mutants (Fig. 6C), and only the *lacA* mutants exhibited slightly recovered growth when treated with proline (Fig. 6B). Furthermore, 20 mM ectoine inhibited growth of the *lon* mutant (Fig. 6B). These data suggested that various compatible solutes have different effects that crosstalk with these genes in compensating for the loss of halotolerance.

**Table 1:** The KCl complementation of cell motility and growth of wild-type and mutated *Halomonas beimenensis*.

Lines	Growth complementation				Cell motility/K^+^-dependent cell motility			
	+Betaine^a^	+Ectoine^b^	+Trehalose^c^	+KCl^d^	−KCl		+KCl	
	15% NaCl	15% NaCl	15% NaCl	15% NaCl	5% NaCl	15% NaCl	5% NaCl	15% NaCl
WT	—	*^e^	—	—	++^f^	—	+++	+
*rfbP*	—	—	—	—	—	—	—	—
*rfbC*	—	—	**	—	—	—	—	—
*lon*	—	—	—	—	—	—	—	—
*smpB*	—	**	*	**	++	—	+++	+
*trkA2*	**	***	—	*	++	—	+++	++
*gdhB*	**	—	—	—	—	—	—	—
*mtnN2*	—	—	***	**	++	—	+++	—
*tatB*	***	*	—	**	++	—	+++	—
*spoT*	***	***	*	—	+	+	+	++
*prkA*	—	—	—	**	++	+	+++	++
*rsbV*	—	—	***	***	++	—	+++	+
*acrR1*	—	—	—	—	++	—	+++	+
*atpC*	—	—	—	**	++	—	+++	+
*nqrA*	—	—	***	*	—	—	+	—
*nadA*	*	—	—	—	+	—	++	—
*lacA*	—	—	***	—	++	—	+	+

^a^20 mM betaine. ^b^20 mM ectoine. ^c^20 mM trehalose. ^d^200 mM KCl. ^e^Significant: *p* < 0.05 (*); *p* < 0.03 (**); *p* < 0.01 (***). ^f^+: increased motility.

In the KCl compensation experiment, 200 mM KCl enhanced the halotolerance of WT bacteria in 15% NaCl (Fig. 6D, and Table 1). Similar to the compensation effects of the compatible solutes, 200 mM KCl fully rescued the mutant phenotype in the *prkA*, *smpB*, *atpC*, *trkA2*, and *rsbV* mutants but had only a slight effect on the *mtnN2*, *tatB*, and *nqrA* mutants (Fig. 6D, and Table 1). By contrast, the growth of the *lon* mutant was repressed in medium containing 15% NaCl with 200 mM KCl, suggesting that KCl has different effects on the various Tn5 mutants (Fig. 6D, and Table 1). In summary, we found that betaine and ectoine exert similar compensatory effects on mutants, whereas KCl and trehalose exert similar compensatory effects on the *mtnN2*, *rsbV*, and *nqrA* mutants (Table 1), implying the existence of type 2 halotolerance in *H. beimenensis*. Nevertheless, these genes belong to the transcription/translation, signaling, and energy production categories.

### Cell motility

**Figure 7:**
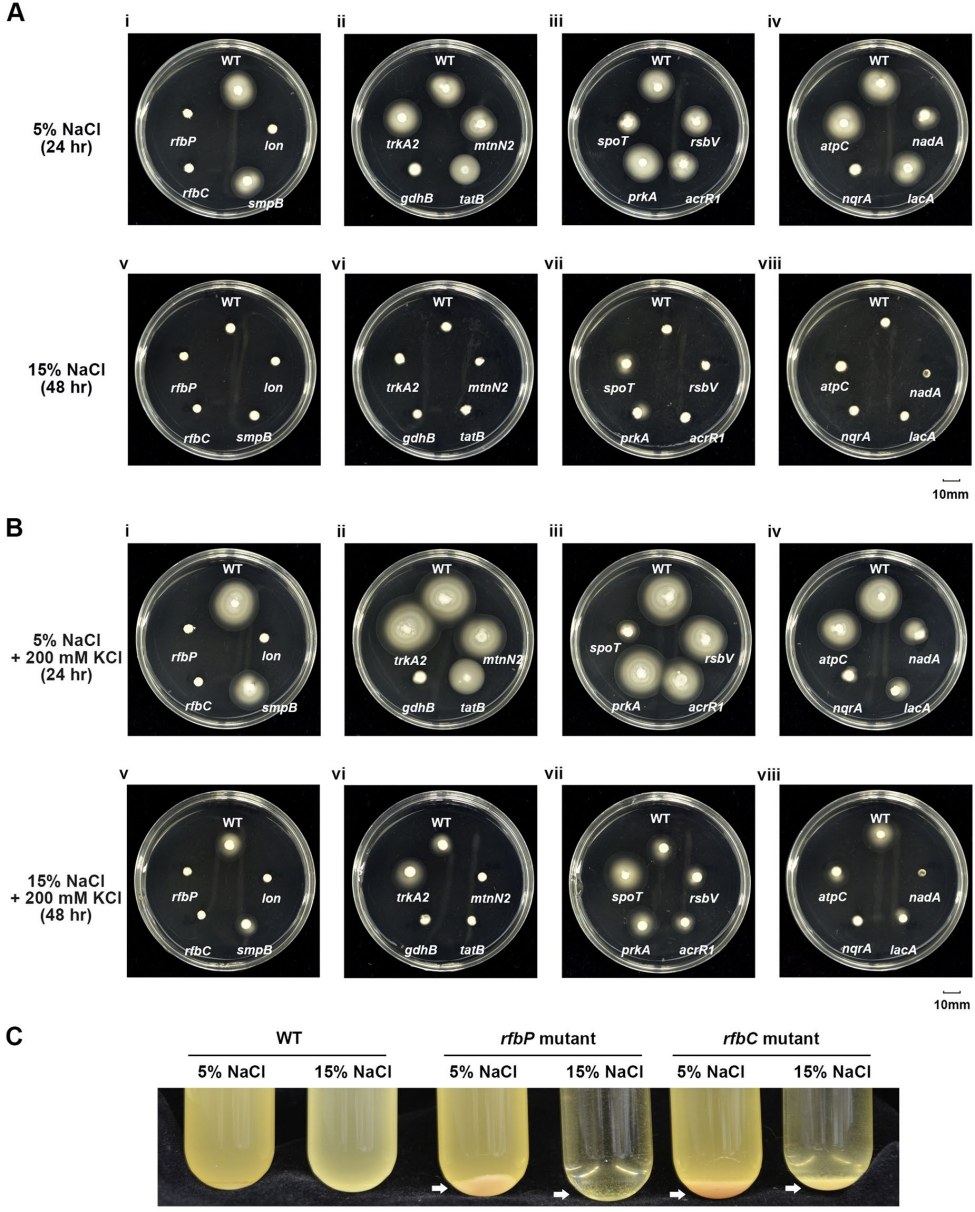
Motility and auto-aggregation phenotype of wild-type and 16 Tn5 mutants of *Halomonas beimenensis*. (**A**) Motility phenotype assay of the wild-type (WT) and 16 Tn5 mutants. (i) to (vi) were assayed on 3% agar plates containing 5% NaCl for 24 h. (v) to (viii) were assayed on 3% agar plates containing 15% NaCl for 48 h. (**B**) Motility phenotype assay of WT bacteria and 16 Tn5 mutants with 200 mM KCl. (i) to (vi) were assayed on 3% agar plates containing 5% NaCl for 24 h. (v)-(viii) were assayed on 3% agar plates containing 15% NaCl for 48 h. (**C**) Auto-aggregation phenotype of the *rfbP* and *rfbC* mutants under different salt conditions.

Bacterial motility is an important behavior used to seek out a suitable environment^30^. The motility of the *rfbP*, *rfbC*, *lon*, *gdhB*, and *nqrA* mutants was completely abolished in 5% and 15% NaCl, suggesting these genes might be positively correlated with motility (Fig. 7A and Table 1). In 15% NaCl, the *spoT* and *prkA* mutants showed only slightly reduced motility, whereas the motility of the other mutants and even the WT was clearly inhibited, suggesting that *spoT* and *prkA*, which are categorized as signaling genes, might be negative regulators of motility under saline stress (Fig. 7B). Notably, the *spoT* mutant showed slightly reduced motility in 5% NaCl but slightly enhanced motility in 15% NaCl (Fig. 7A). Interestingly, the regulatory networks of *spoT* were different between 5% and 15% NaCl, implying that SpoT plays different roles in 5% and 15% NaCl (Fig. 5).

### Potassium-dependent enhancement of cell motility

We wondered whether KCl could also compensate for the loss of motility in these mutants. Compared with treatment with 15% NaCl alone, treatment with 200 mM KCl enhanced and slightly enhanced the motility of WT *H. beimenensis* in 5% NaCl and in 15% NaCl, respectively (Fig. 7A and B), suggesting potassium*-*dependent enhancement of cell motility (Fig. 7A and B, and Table 1). However, the motility of the *rfbP*, *rfbC*, *lon*, *gdhB*, and *nqrA* mutants was not recovered by treatment with 200 mM KCl, suggesting that these genes might be involved in the potassium-dependent enhancement of cell motility (Fig. 7B panels i to iv, and Table 1). To summarize observations regarding KCl compensation and cell motility, KCl enhanced or recovered both the growth and cell motility of the *smpB*, *trkA2*, *rsbV*, and *atpC* mutants in 15% NaCl, whereas KCl recovered cell motility but only slightly recovered the growth of the *mtnN2* and *tatB* mutants in 15% NaCl (Table 1).

### Cell surface properties

Furthermore, mutation of the *rfbP* has been demonstrated to alter cell surface properties, resulting in aggregation of bacteria in liquid culture^31^. Aggregation of the *rfbP* and *rfbC* mutants was observed after the liquid cultures were incubated on the laboratory bench for a few minutes in 5% or 15% NaCl (Fig. 7C). These data agreed with our transcriptome data, indicating that *rfbP* and *rfbC* might play a role in cell wall, cell membrane, and envelope biogenesis (Supplementary Table S3).

## Discussion

NGS has been widely used in many genomics, functional genetics, and epigenetics studies^32^. However, AT-rich repeated sequences within genomic sequences can make it difficult to fill the gaps between contigs^33,34^. The long reads (3,000–37,335 bp/read) obtained by PacBio SMRT sequencing in this study can act as a scaffold to link two contigs^35^. Indeed, our results indicated that the completed genomic sequence of *H. beimenensis* was *de novo* assembled from a single large contig. Therefore, it is possible to obtain complete genomes of prokaryotic bacteria through one-time *de novo* assembly using both short and long reads. Furthermore, we predicted 3,807 genes in the *H. beimenensis* genome, which provided information for whole-transcriptome and Tn5 transposon mutagenesis analyses to identify candidate halotolerance-related genes and study halotolerance mechanisms.

Halophilic bacteria maintain the ionic strength of the cytoplasm at a level equal to that of the external environment by increasing potassium concentrations to overcome saline stress^36^. Nevertheless, most eubacteria exclude ionic solutes and reduce the water activity of the cytoplasm by accumulating organic compatible solutes, which do not disrupt normal cell functions^37^. In *H. beimenensis*, KCl and ectoine slightly enhanced WT bacterial growth but compensated for the loss of halotolerance in particular mutants, suggesting that various compatible solutes exert different effects that exhibit crosstalk with these genes in the compensation of halotolerance. Indeed, NqrA is a subunit of Na^+^-NQR, whereas TrkA2 is involved in the symport of hydrogen ions by the Trk system for potassium uptake^3,9,10^. In *E. coli*, *atpC* encodes subunit ε of ATP synthase, which facilitates the production of a proton gradient across the cell membrane while producing ATP from ADP^24^. Orthologous genes of *nqrA*, *trkA2*, and *atpC* have been found to be associated with energy conversion and inorganic ion transport, and it is therefore not surprising to find that mutation of the three genes results in loss of halotolerance. Based on transcriptome and proteome^20^ profiles, the expression of Na^+^-NQR is up-regulated or increased in high salinity, suggesting sodium ion efflux. Notably, *H. elongata* does not encode an ortholog of *trkA2* of *H. beimenensis*.

In *Halomonas* spp., betaine, ectoine, and proline are important for halotolerance, although certain species also exhibit trehalose uptake, implying that various compatible solutes might facilitate halotolerance in different *Halomonas* species^38–40^. Ectoine synthesis enzymes of *H. elongata* (EctA, EctB, and EctC) are increased in optimal salinity compared with low salinity^20^. However, the levels of these enzymes did not change significantly in *H. beimenensis* in 20% NaCl, suggesting that the amounts of these enzymes were sufficient for ectoine synthesis under high salinity. 2-Oxoglutarate is a precursor of proline that is produced from glutamate by NAD-GDH^25^. However, the application of additional proline did not rescue the growth of the *gdhB* mutant in this study. In addition, NAD is a cofactor in the catalysis of the interconversion of 2-oxoglutarate and glutamate by NAD-GDH, and NadA is involved in the biosynthesis of quinolinic acid (QA), which is a substrate for NAD^+^ biogenesis in *E. coli* and *Salmonella typhimurium* ^25,26^. Therefore, mutations in *nadA* and *gdhB* affect the biosynthesis of compatible solutes.

The identification of new genes involved in other pathways and crosstalk with halotolerance was the main purpose of this study. In transcription, *rsbV* encodes an anti-anti-sigma factor (an anti-sigma factor antagonist) in various species that controls the activation of sigma factor B (SigB) under osmotic stress^41,42^. SigB binds an anti-sigma factor under normal conditions; however, under osmotic stress, the unphosphorylated form of RsbV competes with SigB to bind the anti-sigma factor, resulting in the release of SigB, which then associates with the core RNA polymerase to activate stress response genes^41,42^.

In translation surveillance, *E. coli lon* encodes an ATP-dependent protease that acts as a heat-shock regulon and is involved in selective intracellular proteolysis to control protein quality and maintain cellular homeostasis^43,44^. SmpB forms a ribonucleoprotein complex (tmRNP) with transfer-messenger RNA (tmRNA), elongation factor Tu (EF-Tu), and ribosomal protein S1 to monitor trans-translation^45^. Therefore, SmpB and Lon play roles in translation surveillance and homeostasis, respectively, to control halotolerance.

TatB of *E. coli* is involved in the twin-arginine translocation (TAT) protein export system, and synthesis of H7 flagellin is abolished in a the triple mutant of *TAT* (Δ*tatABC*)^28^. According to our NGS data, 27 of 37 genes associated with flagellar biosynthesis were up-regulated under high-salinity conditions. The question of whether a *tatB* mutation in *H. beimenensis* affects flagellum synthesis will be an interesting topic for future research. In *Pectobacterium atrosepticum* and *Salmonella enterica*, *rfbP* encodes an undecaprenyl-phosphate galactose phosphotransferase, whereas *rfbC* encodes a biosynthetic precursor of O-antigen, which is involved in the synthesis of dTDP-L-rhamnose and influences bacterial motility^31,46^. RfbP and RfbC are related to cell envelope biogenesis; mutation of the corresponding genes could cause deficits in cell envelop develop and, consequently, various dysfunctions, resulting in the misregulation of genes and loss of osmoregulation. The mutation of *rfbP* and *rfbC* in *H. beimenensis*, *S. enterica* and *P. atrosepticum* results in reduced swimming or swarming ability^31,46^.

With respect to quorum sensing, in *V. cholera*, *mtnN* is a 5′-methylthioadenosine nucleosidase that plays a role in S-adenosylmethionine-related quorum sensing pathways, which might facilitate bacterial communication, motility, gene transfer, and secondary metabolites^47,48^. The mutation of *mtnN2* in *H. beimenensis* influences bacterial growth and motility. Notably, no findings have demonstrated a correlation between quorum sensing and halotolerance, and many genes promote *mtnN2* expression in 15% NaCl (Fig. 5B).

*spoT* of *E. coli* encodes a guanosine-3′,5′-(bis)pyrophosphate (ppGpp) synthetase and hydrolase that plays roles in the control of starvation and DNA repair^49,50^. Our data indicated that SpoT plays significant roles in the regulation of compatible solutes and cell motility. *E. coli lacA* encodes a galactoside *O*-acetyltransferase that is associated with the lactose operon *lacZYA*, which is involved in carbohydrate metabolism^51,52^. This study is the first to report that LacA influences halotolerance in *H. beimenensis*.

Finally, the functions of PrkA and AcrR1 are unknown; however, our bioinformatics data suggest that PrkA is involved in signaling and that AcrR1 is involved in translation. *prkA* is down-regulated under high-salinity conditions in *H. beimenensis* and *M. alhagi* ^53^. However, the *prkA* mutant of *H. beimenensis* shows lower tolerance to saline stress, whereas the *M. alhagi prkA* mutant exhibits tolerance^53^, suggesting that PrkA is associated with the regulation of salt stress but exhibits different functions in different bacterial species. Moreover, AcrA and AcrB of *E. coli* are positive regulators of osmotic stress, whereas AcrR is a repressor of the *acrA* and *acrB* genes^54^. However, the *acrR1* mutant of *H. beimenensis* lost its saline adaptation ability, implying the existence of unknown mechanism for AcrR1-regulated halotolerance.

Figure 5B summarizes gene functions and interactions in halotolerance and cell motility. Based on the network associated with treatment with 15% NaCl, a halotolerance signal could be communicated via quorum sensing by *mtnN2* expression triggered by other functions, such as cell motility, energy and ion transport. Na^+^-NQR and ATPase, which are involved in sodium pumps and energy production, respectively, are negatively regulated by the flagellum. Moreover, NadA and GdhB could be involved in compatible solute production for saline adaptation. Motility assays indicated that PrkA and SpoT negatively regulate cell motility but that genes in the cell motility and compatible solute categories, among others, promote cell motility. Interestingly, these cell motility-related genes exhibit high overlap with the potassium-dependent enhancement of motility. Finally, compatible solute production and response genes were identified through bioinformatics and experimental approaches in this study. The complete genome of *H. beimenensis* and the 16 mutants can be employed as a model system to further investigate mechanisms of halotolerance in the future. From an industry perspective, *H. beimenensis* became an attractive strain in fermentation because of underlying mechanisms of halotolerance. Moreover, this study can be useful in informing strain selection for fermentation application.

## Methods

### Bacterial strain and growth conditions

The *H. beimenensis* NTU-111 strain used in this study was originally isolated from brine samples from the Beimen saltern in southern Taiwan^22^. *H. beimenensis* was grown in basal medium containing 5 g/L yeast extract (Bacto, BD), 5 g/L Casamino acids (Bacto, BD) and 5 g/L MgSO_4_·7H_2_O (Wako), pH 7.5. To determine the optimal growth conditions for *H. beimenensis*, the bacteria were grown in basal medium with various concentrations of NaCl, including 0%, 5%, 10%, 15%, 20%, and 25% NaCl (w/v), and were incubated at 37 °C with shaking at 220 rpm. The concentration of *H. beimenensis* (OD600) was monitored by a spectrophotometer (Libra S4, Biochrom) every 6 h until 72 h with three replicates.

### Genomic DNA and total RNA extraction

*H. beimenensis* was grown in basal medium with 5% NaCl at 37 °C for 16 h (OD_600_ = 2.0), and the genomic DNA was extracted using a Qiagen Gentra Puregene Kit (Qiagen) according to the manufacturer’s instructions. For total RNAs purification, *H. beimenensis* cells (OD_600_ = 2.0) were grown in medium containing 5%, 15%, and 20% NaCl, and RNA was extracted using a Total RNA Purification Kit (Geneaid) according to the manufacturer’s instructions.

### Phylogenetic tree analysis

For phylogenetic tree analysis, MEGA v7 was used to compare 16 S rRNA sequences of the *Halomonadaceae* family (Supplementary material) were compared via the neighbor-joining method with 1,000 bootstrap replicates^55^. Here, *Escherichia coli* (U00006) and *Pseudomonas aeruginosa* (X06684) were selected as out groups.

### Genomic DNA and whole-transcriptome sequencing

The whole genome sequencing of *H. beimenensis* was performed on the MiSeq (2 × 300) paired-end sequencing (Illumina) and SMRT (20k) (PacBio) platforms (Genomics, BioSci & Tech Co.). The whole-transcriptome sequencing was performed using MiSeq (2 × 300) paired-end strand-specific sequencing (Illumina) (Genomics, BioSci & Tech Co.). The raw reads reported in this paper are available in the NCBI Short Read Archive under accession numbers SRR5572247 (genomic DNA for PacBio), SRR5572216 (genomic DNA for Illumina), SRR5572264 (transcriptome for 5% NaCl), and SRR5572268 (transcriptome for 20% NaCl).

### Genomic DNA assembly, gene prediction, annotation, and gene comparison

The whole-genome sequence of *H. beimenensis* was *de novo* assembled by the SPAdes Genome Assembler v3.5.0 using hybrid assembly of the Illumina short reads and the PacBio long reads with default parameters^23^. The web-based software Rapid Annotation using Subsystem Technology (RAST; version 2.0) on the PARTIC platform (www.patricbrc.org/portal/portal/patric/Home) was used for gene prediction and annotation^56,57^. GC content was also calculated with the PARTIC platform. Transfer RNAs and rRNAs were predicted with tRNAscan^58^ and RNAmmer^59^, respectively. The whole-genome graphical representation was constructed using CIRCOS (version 0.67–7)^60^. For GO, gene sequences were analyzed using BLAST2GO (version 3.3.5) with default settings^61^. For COG analysis, gene sequences were analyzed using NCBI’s COG database (version 2014) (www.ncbi.nlm.nih.gov/COG/)^62^. For analysis of the gain and loss of genes in *H. beimenensis*, the coding genes of four *Halomonas* spp., *H*. *campaniensis*, *H*. *chromatireducens*, *H*. *elongata*, and *H*. *huangheensis* were used for ortholog group clustering by OrthoMCL (orthomcl.org/orthomcl/) (Li *et al*., 2003).

### Transcriptome data processing and differential gene expression analysis

For gene expression analysis, clean transcriptomic reads from the 5% and 20% NaCl samples were aligned to the whole genome sequence of *H. beimenensis* using Bowtie2 software (version 2.2.5)^63^. The transcript expression levels were represented using FPKM values evaluated with eXpress (version 1.5.1)^64^. Genes were considered to be differentially expressed if the absolute value of the log_2_FC in FPKM exceeded 2.

### Tn5 transposon mutagenesis

Mutagenesis was performed using the EZ-Tn5^TM^ < R6Kγori/KAN-2 > Tnp Transposome^TM^ Kit (Epicentre) to generate mutants according to the manufacturer’s protocol. Electrocompetent *H. beimenensis* cells were prepared through Choi’s approach, with some modifications^65^. The Tn5 transposon was introduced into the *H. beimenensis* cells in a 2-mm cuvette with 2.5 kV for 5 ms. After electroporation, the cells were suspended in 900 mL of basal medium containing 5% (w/v) NaCl and recovered for 1 h at 37 °C with shaking at 220 rpm and then plated the electroporated cells on basal medium agar containing 5% NaCl and 50 µg/mL kanamycin. The Tn5-transformed colonies were subcultured in basal medium containing 5% or 15% NaCl for halotolerance evaluation.

Mutants that exhibited decreased or lost halotolerance in 15% NaCl were selected. To identify Tn5 insertion positions, the genomic DNA from the mutants was digested with *Hyp*99I, *Eag*I, *Ban*I, *Bgl*II, *Bsa*HI, *Sfo*I, *Hyp*99I, or *Bsa*I, and then self-ligated and transformed into TransforMax^TM^ EC100D^TM^ pir + Electrocompetent *E. coli* cells (Epicentre) that were plated on LB agar containing 50 µg/mL kanamycin for recovery of the Tn5 insertion fragment. Rescued plasmid DNA was extracted and sequenced using the EZ-Tn5 <R6Kγori/KAN-2> transposon-specific primers.

### Validation of gene expression

qRT-PCR was used to validate gene expression in mutants and WT *H. beimenensis*. Primers were designed using Primer3 and the BLAST tools available from NCBI (www.ncbi.nlm.nih.gov/tools/primer-blast/), and the primer sequences are listed in Supplementary Table S5.

### Complementation of the Tn5 mutants and evaluation of cell motility

The chemical complementation of the Tn5 mutants was performed by adding 200 mM KCl, 20 mM betaine, 20 mM ectoine, 20 mM glutamate, 20 mM proline, or 20 mM trehalose to basal medium containing 5% or 15% NaCl. To analyze cell motility, 20 µL of bacterial cells (WT or mutants) (OD600 = 2.0) were incubated at 37 °C on semi-solid basal medium agar plates (3% agar) with 5% or 15% NaCl. For the 5% NaCl condition, the bacteria were incubated up to 24 h, whereas the bacteria were incubated up to 48 h for the 15% condition. The diameter of each colony was determined.

## Electronic supplementary material

Supplementary Material

Supplementary Table 1

Supplementary Table 2

Supplementary Table 3
