# Supplementary material for: Revealing the Saline Adaptation Strategies of the Halophilic Bacterium Halomonas beimenensis through High-throughput Omics and Transposon Mutagenesis Approaches
Source: Sci Rep. 2017 Oct 12;7:13037. doi: 10.1038/s41598-017-13450-9 (PMC5638851; doi:10.1038/s41598-017-13450-9)
Supplement: Supplementary file 1 — Supplementary Material [file 41598_2017_13450_MOESM1_ESM.pdf]

Revealing the Saline Adaptation Strategies of the Halophilic Bacterium *Halomonas beimenensis* through High-throughput Omics and Transposon Mutagenesis Approaches

Yan-Huey Chen[1,2], Chia-Wei Lu[2], Yuan-Tay Shyu[1,2,6], Shih-Shun Lin[2,3,4,5,6]

[1]Department of Horticulture and Landscape Architecture, National Taiwan University, Taipei, Taiwan 106.
[2]Institute of Biotechnology, National Taiwan University, Taipei, Taiwan 106.
[3]Agricultural Biotechnology Research Center, Academia Sinica, Taipei, Taiwan 115.
[4]Center of Biotechnology, National Taiwan University, Taipei, Taiwan 106.
[5]National Center for High-Performance Computing, National Applied Research Laboratories, Hsinchu, Taiwan 300.
[6]Correspondence: Shih-Shun Lin; Email: linss01@ntu.edu.tw; Yuan-Tay Shyu; Email: tedshyu@ntu.edu.tw.

Supplementary Material

Gene features of *H. beimenensis*

A total of 3,807 identified coding sequences (CDSs) were spread across the positive strand (1,868 CDSs) and negative strand (1,939 CDSs) (Fig. 2). The average G+C content was 68.4%, and the GC skew indicated that the replication origin and replication terminus were located between 4,008 kb and 1,908 kb (Fig. 2). Based on the annotation of genes from the NCBI nr database, 3,098 unigenes were assigned to three categories of the clusters of Gene Ontology, cellular components (579 genes), molecular functions (1,504 genes), and biological processes (1,015 genes), which contained 119 sub-categories (Supplementary Fig. S2). The genes in the cellular components category were assigned to sub-categories that included "cytoplasm" (142 genes), "membrane" (51 genes), and "cell" (27 genes), among others (Supplementary Fig. S4). In addition, the genes in the molecular functions category were assigned to sub-categories that included "DNA binding" (324 genes), "oxidoreductase activity" (237 genes), and "ATPase activity cell" (137 genes), among others (Supplementary Fig. S2). Moreover, the genes in the biological processes category were assigned to sub-categories that included "small molecule metabolic process" (356 genes), "transport" (101 genes), and "transmembrane transport" (57 genes), among others (Supplementary Fig. S2). Based on the sequence homology, a total of 3,786 genes were mapped to 23 different clusters of orthologous groups of proteins (COG) categories (Supplementary Fig. S3). The top three mapped COG categories were "Amino acid transport and metabolism" (355 genes), "Energy production and conversion" (289 genes), and "Translation, ribosomal structure, and biogenesis" (262 genes) (Supplementary Fig. S3). Overall, these 3 categories accounted for 23.93% (906 of 3,303 genes) of the annotated COG functional dataset (Supplementary Fig. S3).

Transcriptome profiles of *H. beimenensis*

The transcriptome profiles of *H. beimenensis* grown in 5% and 20% NaCl were used to identified differentially expressed genes (DEGs). The transcriptome reads obtained from bacteria grown in 5% (5,070,041 reads) and 20% (6,154,708 reads) NaCl were compared to the genomic sequence to determine the mapping rate and to perform FPKM calculations. The mapped transcriptome reads are shown in the genome map (Fig. 2). The 4th and 6th circles represent the transcriptome read counts on the + and – strands in the 5% NaCl condition, respectively, whereas the 5th and 7th circles represent the transcriptome read counts on the +

and – strands in the 20% NaCl condition, respectively (Fig. 2). Our data indicated that approximately 98.66% (5% NaCl) and 96.02% (20% NaCl) of the transcriptome reads were mapped to the genomic sequence. Moreover, approximately 53.61% (in 5% NaCl) and 74.59% (in 20% NaCl) of the reads mapped to CDSs, whereas 40.05% (5% NaCl) and 21.43% (20% NaCl) of the reads mapped to intergenic regions, implying that intergenic regions of the genome can also generate RNA transcripts. The average TPM (transcripts per million reads) of CDSs was approximately 594,952.57 and 811,185.16 in 5% and 20% NaCl, respectively. However, the average TPM of the intergenic regions was approximately 405,047.43 and 188,814.83 in 5% and 20% NaCl, respectively (Fig. 3A). These data suggested that CDSs exhibit higher transcriptional expression than intergenic regions and that CDS transcription was enhanced in 20% NaCl.

We identified 614 DEGs with a $\log_2$ fold-change ($\log_2$FC) in FPKM > 2 between 5% and 20% NaCl groups (Supplementary Table S3). In bacteria grown in 20% NaCl, 268 genes were up-regulated, and 346 genes were down-regulated, compared with bacteria grown in 5% NaCl condition (Fig. 3B, and Supplementary Table S3). The up-regulated genes were classified into 20 COG functional categories (Supplementary Fig. S2), the top five of which were “cell motility” (34 genes), “energy production and conversion” (30 genes), “translation, ribosomal structure and biogenesis” (28 genes), “inorganic ion transport and metabolism” (22 genes), and “carbohydrate transport and metabolism” (19 genes) (Fig. 3C and Supplementary Table S3). These data indicated that many flagellar-related *H. beimenensis* genes were up-regulated in the 20% NaCl condition (Fig. 3E). In addition, the genes that were down-regulated in 20% NaCl were classified into 20 COG functional categories (Supplementary Fig. S2), the top five of which were “amino acid transport and metabolism” (42 genes), “lipid transport and metabolism” (37 genes), “energy production and conversion” (30 genes), “signal transduction mechanisms” (24 genes), and “transcription” (20 genes) (Supplementary Table S3). Notably, many acetyl-CoA biosynthesis genes function in lipid metabolism pathways (Fig. 3C). The top Gene Ontology (GO) categories of the gene up-regulated in 20% NaCl were “small molecule metabolic process” (30 genes), “oxidoreductase activity” (27 genes), “structural constituent of ribosome” (23 genes), and “cell motility” (16 genes), while the top categories of the genes down-regulated in 20% NaCl were “DNA binding” (30 genes), “oxidoreductase activity” (27 genes), “small molecule metabolic process” (26 genes) (Fig. 3D, and Supplementary Table S3). Therefore, these data suggested that gene regulation in high salt conditions involves many functional biosynthesis pathways, especially in cell motility, and energy metabolism.

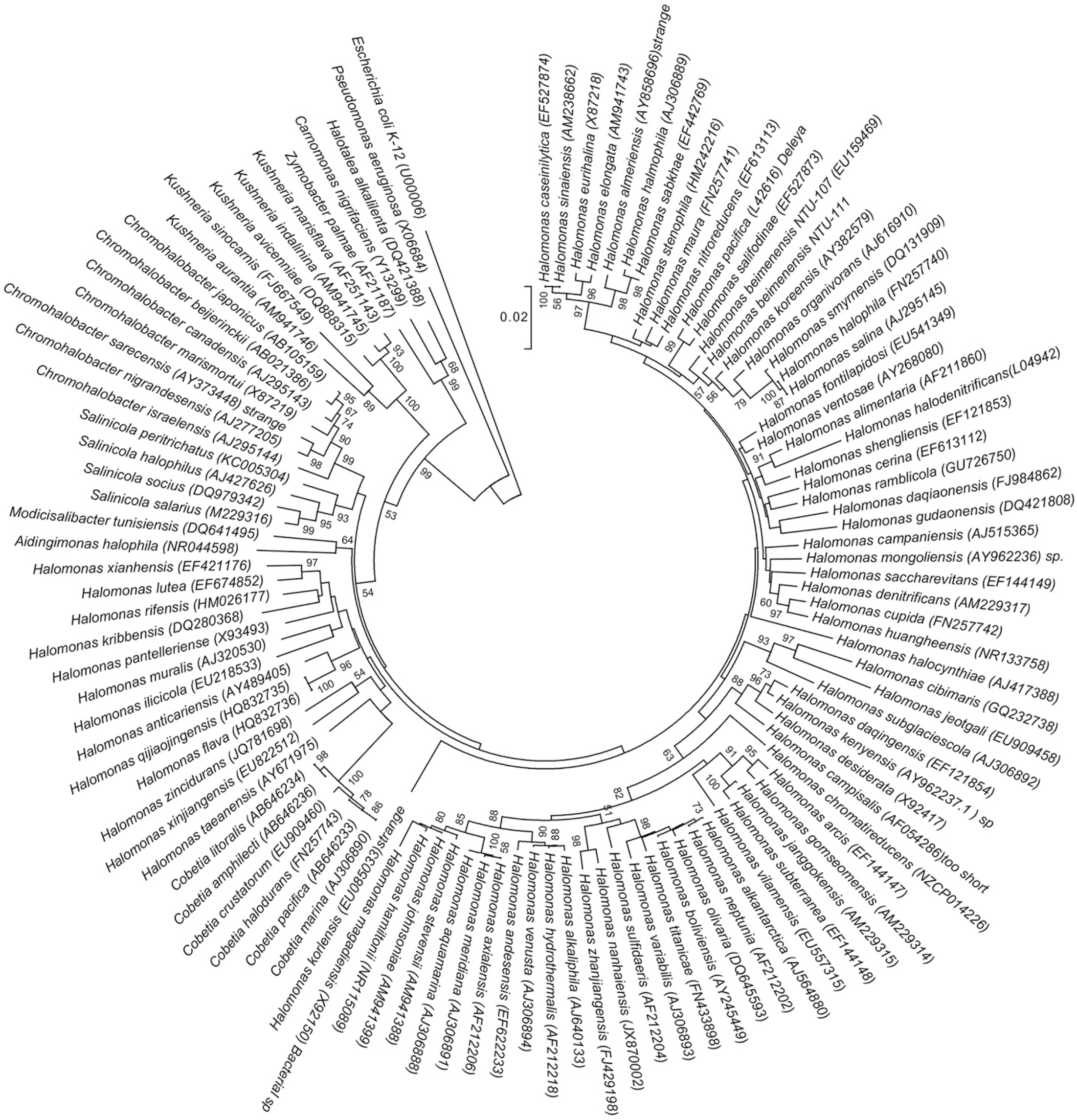

Supplementary Figure S1. Neighbor-joining phylogenetic tree of the family *Halomonadaceae* based on 16S rRNA. Bootstrap values were calculated from 1,000 samplings, and values > 50% are shown. Bar, 0.02 substitutions per nucleotide position.

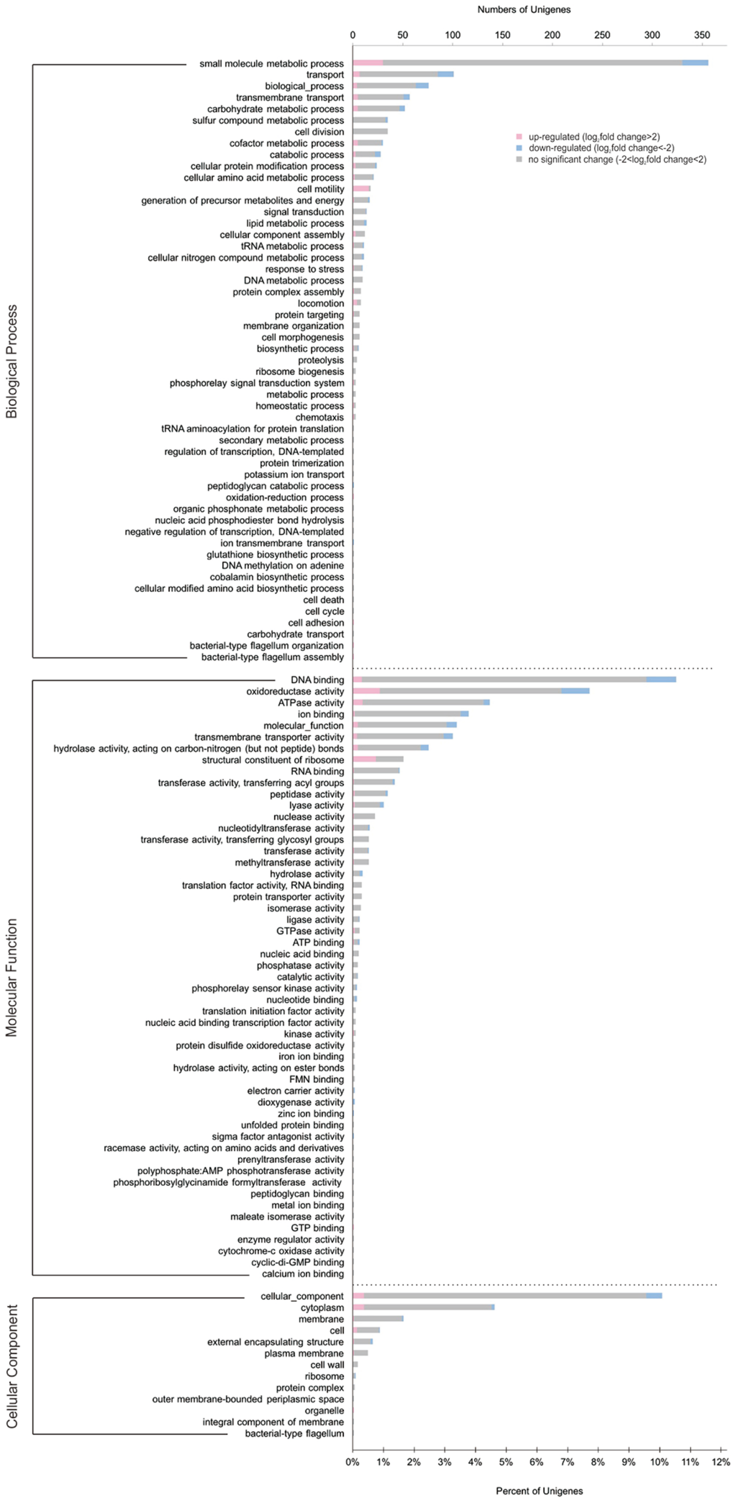

Supplementary Figure S2. The clusters of Gene Ontology (GO) categories indicated for the differentially expressed genes (DEGs) of *Halomonas beimenensis*.

The upper and lower X-axes indicate the numbers and percentage of genes in a category, respectively. Up-regulated DEGs [log2 fold-change (log$_2$FC) of FPKM > 2] are represented by pink bars; the down-regulated DEGs (log$_2$FC of FPKM < -2) are represented by blue bars; gray bars represent genes with no significant change (-2 < log$_2$FC of FPKM < 2).

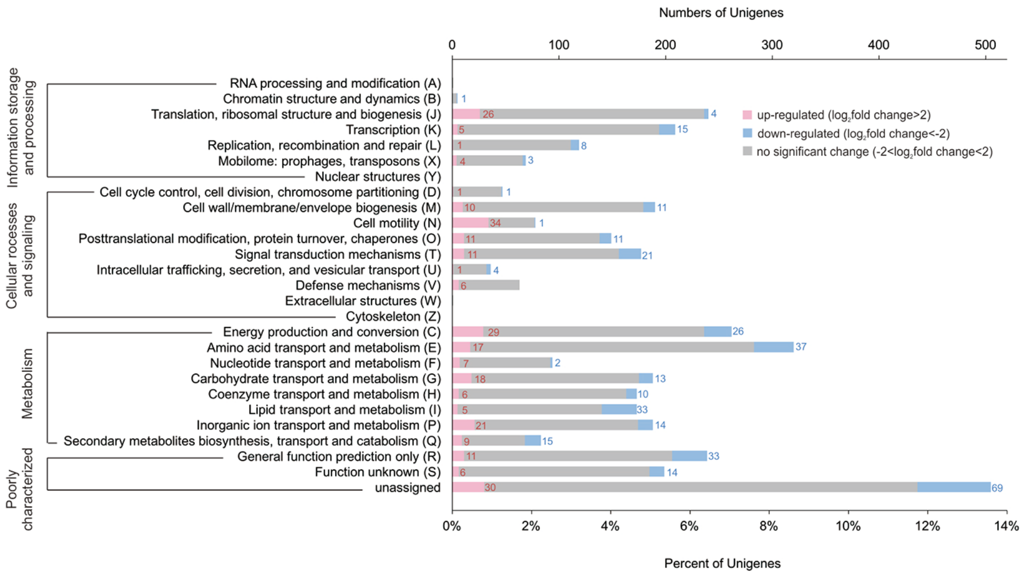

Supplementary Figure S3. The clusters of orthologous groups (COG) categories indicated for the differentially expressed genes (DEGs) of *Halomonas beimenensis*.

The upper and lower X-axes indicate the numbers and percentage of genes in a category, respectively. The up-regulated DEGs [$\log_2$ fold-change ($\log_2$FC) of FPKM > 2] are represented with pink bars; the down-regulated DEGs ($\log_2$FC of FPKM < -2) are represented with blue bars; gray bars represent genes showing no significant change (-2 < $\log_2$FC of FPKM < 2).

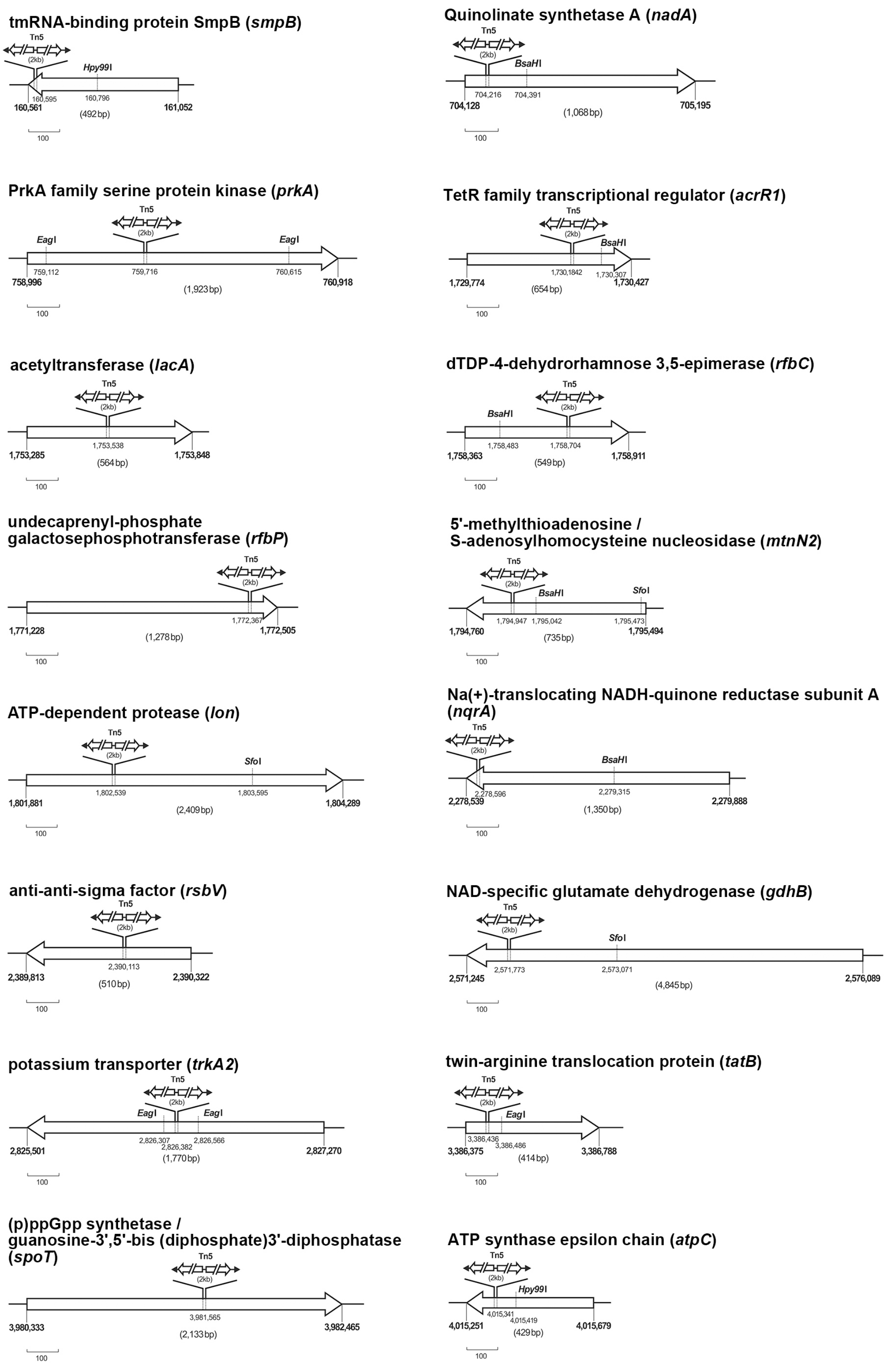

Supplementary Figure S4. The Tn5 transposon insertion site in mutant *Halomonas beimenensis* lines.

Supplementary Table S4. The significant *p-value* of each gene expression analysis.

Gene	down-regulation	up-regulation
rsbV	7×10^{-14}	3.57×10^{-2}
trkA2	3.35×10^{-6}	1×10^{-2}
smpB	9×10^{-7}	7×10^{-4}
nadA	1×10^{-6}	4×10^{-2}
acrR1	3.66×10^{-7}	3×10^{-5}
mtnN2	4.5×10^{-4}	1×10^{-3}
rfbP	1.1×10^{-6}	1×10^{-3}
rfbC	4.5×10^{-5}	3×10^{-2}
spoT	1×10^{-3}	7×10^{-3}
lacA	1×10^{-5}	1.5×10^{-3}
lon	1×10^{-10}	2×10^{-3}
prkA	1.5×10^{-6}	1×10^{-3}
tatB	1.5×10^{-5}	2.5×10^{-3}
atpC	4×10^{-4}	4×10^{-3}
nqrA	4×10^{-4}	2.5×10^{-3}
gdhB	1×10^{-7}	1×10^{-3}

Supplementary Table S5. Primer set for qRT-PCR analysis.

Primer Name	Sequence (5′ to 3′)
BEIMEN_16S1003	AGAGATGGATTGGTGCCTTC
BEIMEN_16S1154	CGGCAGTCTCCCTAGAGTTC
BEIMEN_lon2064	CATGGTCACCGCCATGATCT
BEIMEN_lon2213	GTCTTTATACCACCGCGCCT
BEIMEN_mtnN473	ACCTGGTCGCGAAGACCC
BEIMEN_mtnN576	CACGAAGGGACAGCCATAGA
BEIMEN_rfbP387	TCGCAACTTCCGCATCTACA
BEIMEN_rfbP523	GCAACTCATCAAGGCGTGTC
BEIMEN_rfbP313	AATCAGCACCAGCTATGGGT
BEIMEN_rfbP425	CTTTCGCTCTCAGGGGCATA
BEIMEN_lacA97	AGCGGTAGCGCAATGGTAAA
BEIMEN_lacA224	CCGATCGTCACACCAGGAAA
BEIMEN_acrR387	CTTCATTCGCGAGGAGTACG
BEIMEN_acrR509	ATCACCGACCCGAGGATGA
BEIMEN_prkA770	TCGTCGAGATGTTCAAGGCG
BEIMEN_prkA937	TGTTGCGATTGTTGCGGAAG
BEIMEN_nadA769	GATCTCAAGGGCCTGTACCC
BEIMEN_nadA819	GATCAGCTTGTCATGGGGCA
BEIMEN_smpB351	GCTCTACTGGAAGGGCAACA
BEIMEN_smpB467	ACGGTTTTGTTCGCGCATGA
BEIMEN_atpC3	GACGAAAACCTTCAAGTGCGA
BEIMEN_atpC89	CACCCCGAGATCCCCCA
BEIMEN_spoT753	GAAGTCGTTCGCCGAGATCA
BEIMEN_spoT860	ACCGGCTTGTAGAGGTTGTG
BEIMEN_tatB2	TGTTCGACATCGGCTTCCTC
BEIMEN_tatB133	CGGACACGGTACGCTTGAT
BEIMEN_trkA1632	CATCGGCTACCAGACCAACC
BEIMEN_trkA1760	CAGACGATCGGAATCAGCCC
BEIMEN_gdhB4273	GATGCCATCGACTACTTCGC
BEIMEN_gdhB4377	CTCCTGGCGACGAACCTC
BEIMEN_rsbV130	CCAGGACTTGAAGCGGTGAT
BEIMEN_rsbV263	TTGTCGACGACGATGGTGGG
BEIMEN_nqrA558	CGAAGGCAAGGTGTTCCTGT
BEIMEN_nqrA696	TACGGGCGAGAGATGATGGA
